# Supplementary material for: Unravelling the transcriptome of the human tuberculosis lesion and its clinical implications
Source: Nat Commun. 2025 May 30;16:5028. doi: 10.1038/s41467-025-60255-w (PMC12125219; doi:10.1038/s41467-025-60255-w)
Supplement: Supplementary file 1 — Supplementary Information [file 41467_2025_60255_MOESM1_ESM.pdf]

Supplementary Information

Unravelling the transcriptome of the human tuberculosis lesion and its clinical implications

Kaori L. Fonseca[1,2], Juan José Lozano[3,4], Albert Despuig[1,2], Dominic Habgood-Coote[5], Julia Sidorova[3,4], Diego Aznar[1,6], Lilibeth Arias[1,2], Álvaro Del Río-Álvarez[7,8,9], Juan Carrillo-Reixach[7,8,9], Aaron Goff[10], Leticia Muraro Wildner[10], Shota Gogishvili[11], Keti Nikolaishvili[11], Natalia Shubladze[11], Zaza Avaliani[11,12], Gustavo Tapia[13,14], Paula Rodríguez-Martínez[13,14], Pere-Joan Cardona[1,2,6,15], Federico Martinón-Torres[2,16,17,18], Antonio Salas[2,18,19,20], Alberto Gómez-Carballa[2,18,19,20], Carolina Armengol[7,8,9], Simon J Waddell[10], Myrsini Kaforou[5,21], Anne O'Garra[22,23], Sergo Vashakidze[11,24]*, Cristina Vilaplana[1,2,6,15,25,*,&]

* These authors jointly supervised this work: Sergo Vashakidze and Cristina Vilaplana
& Correspondence and requests should be addressed to C.V (email: cvilaplana@igtp.cat)

a

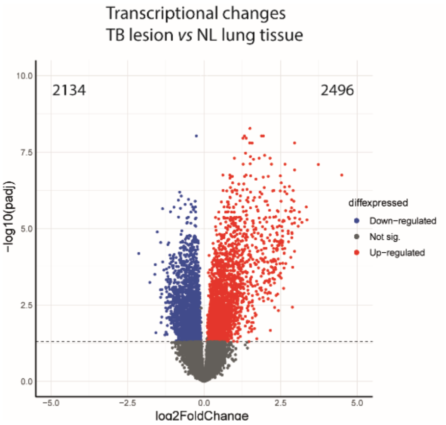

b

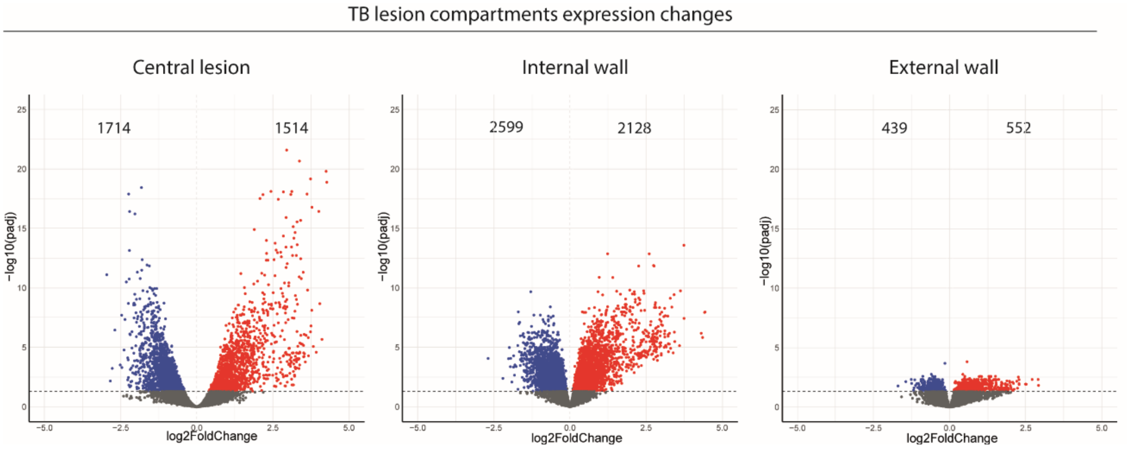

Supplementary Figure 1. Differential expression analysis of human TB lesion and separated compartments relative to non-lesional lung tissue. Panel a) Volcano plot depicts differentially expressed genes (DEGs) between TB lesion and non-lesional (NL) lung tissue. **Panel b)** Volcano plot depicts DEGs between Central, Internal and External compartments and the non-lesional (NL) lung tissue. Genes with the adjusted p-value ≤ 0.05 were considered significantly down-regulated when the log2 fold change was < 0.1 (blue) and up-regulated when log2fold change > 0.1 (red). Source data are provided as a Source Data file.

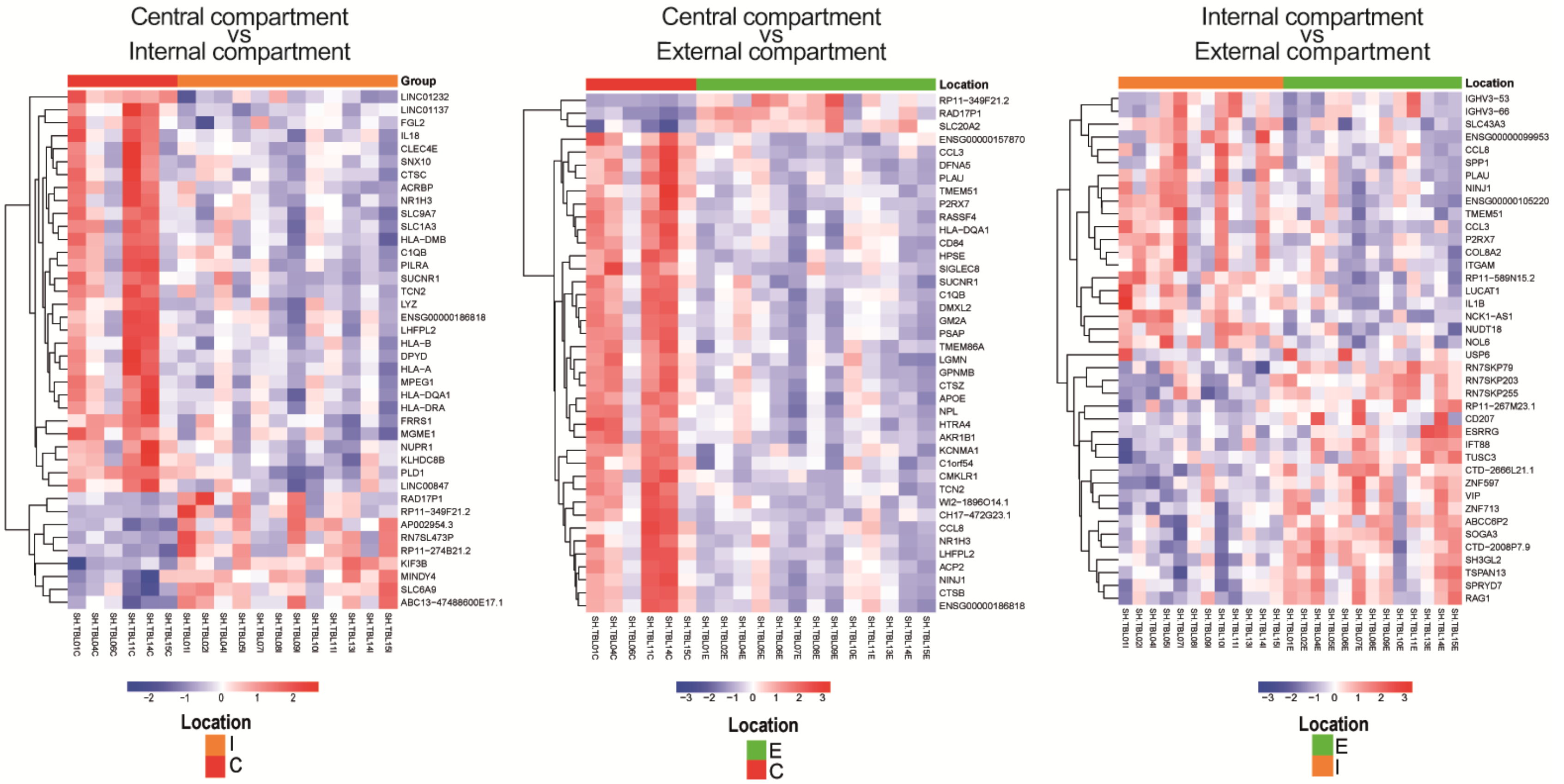

Supplementary Figure 2. Differential expressions of human TB lesion compartments between each other. Heatmaps showing differences in the top 40 ranked genes from DESeq2 with $p<0.05$ by separately comparing the central (C) to the internal (I) compartment, central (C) to the external (E) compartment and the internal (I) to the external (E) compartment (44 paired samples from 13 patients). The intensity of each colour denotes the standardized ratio between each value and the average expression of each gene across all samples. Red pixels correspond to an increased abundance of mRNA in the indicated sample, whereas blue pixels indicate decreased mRNA levels. Source data are provided as a Source Data file.

a

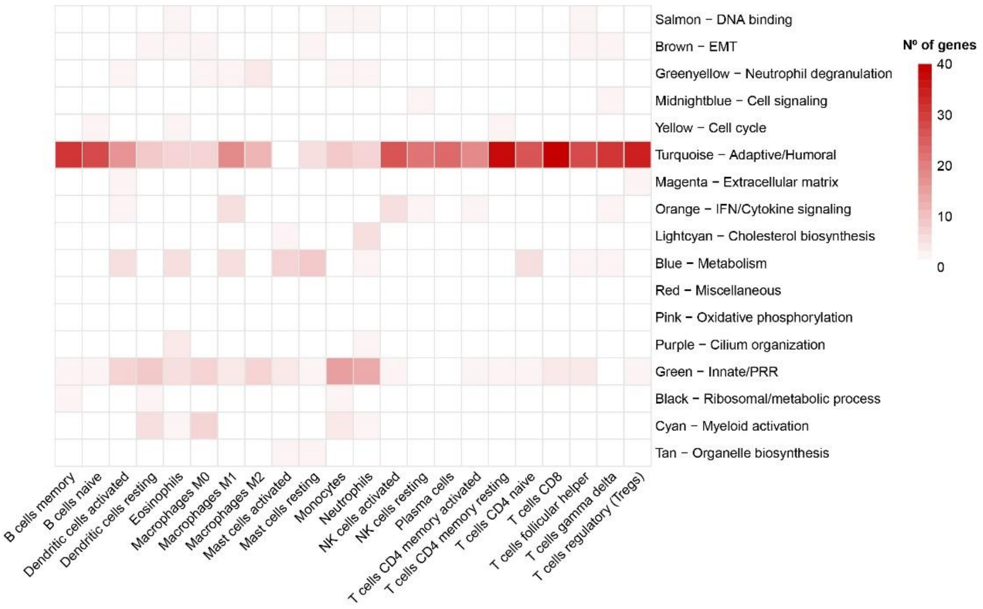

b

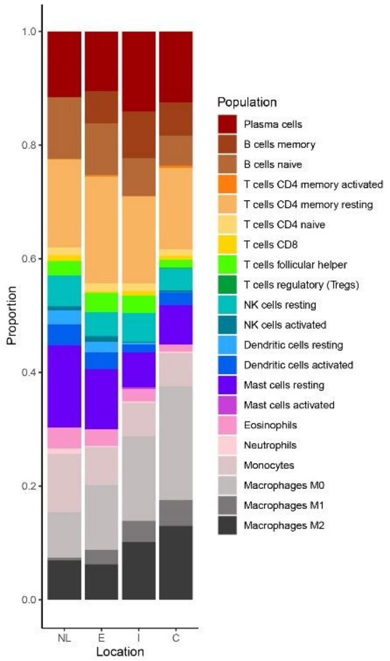

Supplementary Figure 3. Immune populations associated with modular transcriptomic signature in the TB lesion and their distribution among TB lesion compartments. Panel a depicts the distribution of the LM22 signature and the corresponding immune populations among the TB lesion modules. Color intensity represents the number of LM22 genes associated with the corresponding immune population present in each TB lesion module. EMT = Epithelial to Mesenchymal Transition; PRR = Pattern Recognition Receptors. **Panel b** shows the proportional distribution of LM22 immune populations across non-lesional tissue (NL) and TB lesion external (E), internal (I), and central (C) compartments, as obtained from CIBERSORT deconvolution. Source data are provided as a Source Data file.

a b

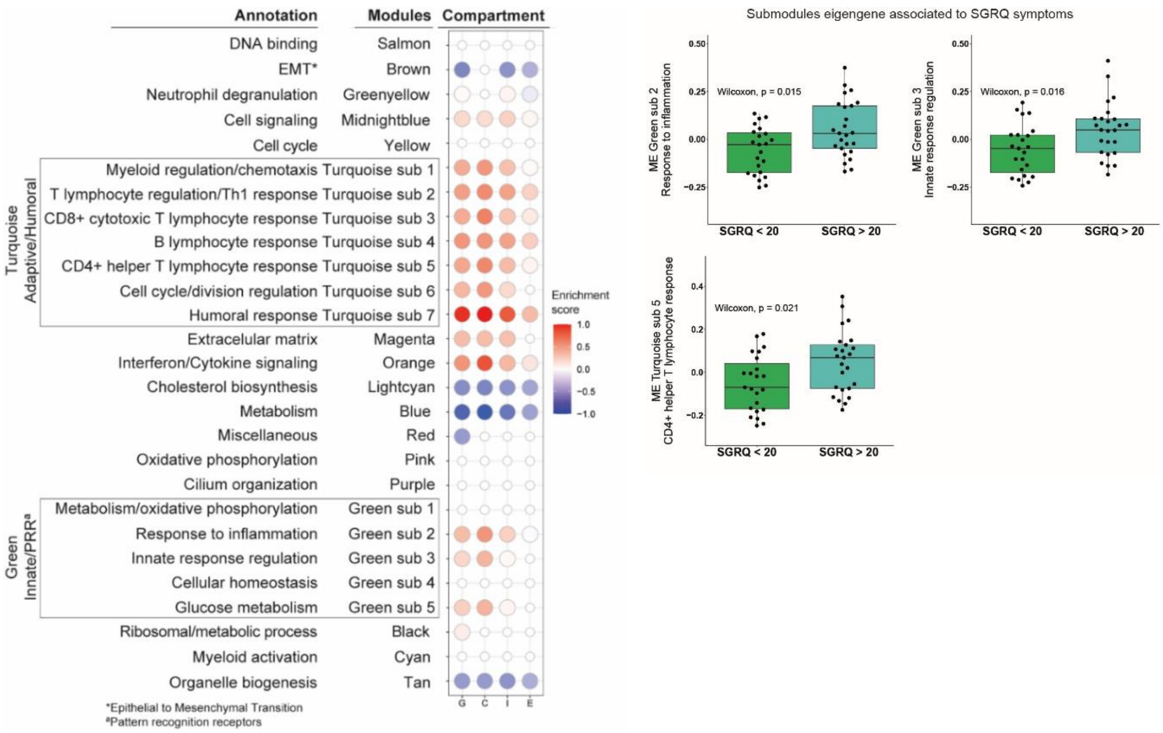

Supplementary Figure 4. Enrichment by TB lesion compartment and association with clinical surrogates of sub-modules resulting from the expansion of immune-related modules. Panel a depicts the enrichment by TB lesion compartment of the modular transcriptomic signature, including the sub-modules resulting from the expansion of the adaptive/humoral and innate/PRR modules. Fold enrichment scores derived from using QuSAGE are depicted, with red and blue indicating modules over or under expressed compared to the control. Only modules with fold enrichment FDR < 0.1 were considered significant. **Panel b** shows the TB individuals' stratification according to SGRQ symptom score (low impact if SGRQ < 20 with n=23 or high impact if SGRQ > 20 with n=25), and the significant association (p<0.05) using their corresponding derived WGCNA eigengene (ME) for the adaptive/humoral and innate/PRR sub-modules. Data are represented as median with an interquartile range (IQR). Boxplots show minimum and maximum values, the interquartile range (IQR, 25th to 75th percentile), and the whiskers representing 1.5 times the interquartile range. Outliers are indicated as individual points outside the whiskers. Statistical analysis was performed by applying the two-sided Wilcoxon-rank sum test. Source data are provided as a Source Data file.

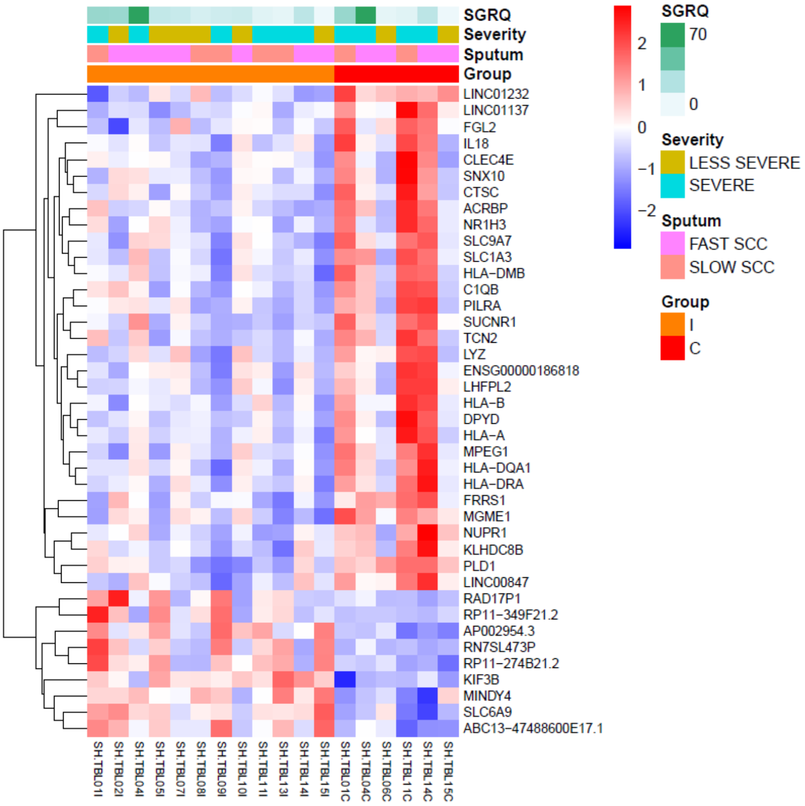

Supplementary Figure 5. Clustered gene expression comparison between the internal and the central compartments including annotation by clinical surrogates of disease severity. The heatmap depicts the top 40 DEGs ranked by p-value comparing the internal vs the central expression profiles. Clustering corresponds to the TB lesion compartment and is accompanied by stratification of fast and slow sputum culture converters, TB severity measured using the Saint George's Respiratory Questionnaire (SGRQ) symptom score and the score itself. The intensity of each colour denotes the standardized ratio between each value and the average expression of each gene across all samples. Red pixels correspond to an increased abundance of mRNA in the indicated sample, whereas blue pixels indicate decreased mRNA levels. Source data are provided as a Source Data file.

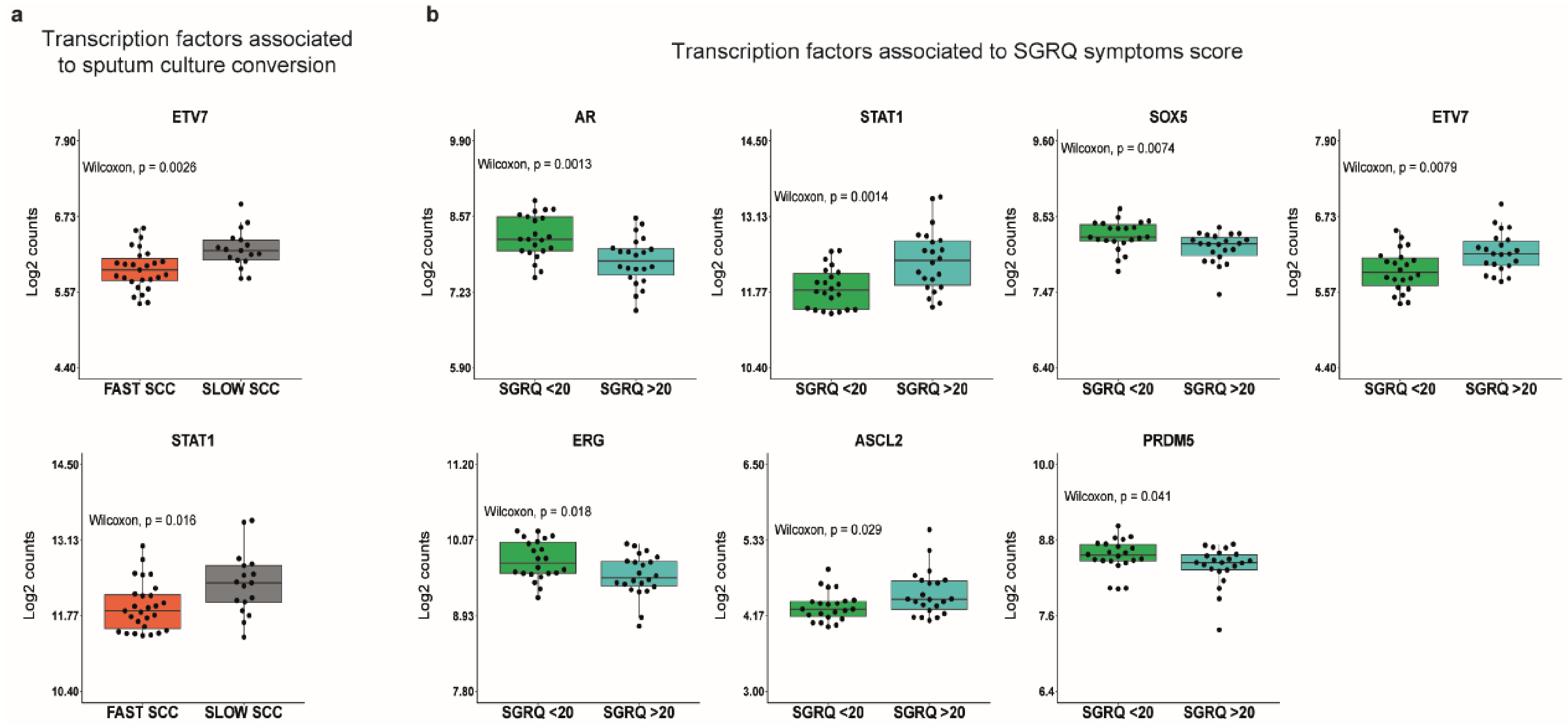

Supplementary Figure 6. Transcription factors associated with clinical surrogates of disease severity. Panel a and **b** depict box plots representing the mean differences for each transcription factor expression (log2 normalized counts) between fast (n=27) or slow (n=17) sputum culture converters, and less severe and more severe disease; based on SGRQ symptom score (low impact if SGRQ < 20 with n=22 or high impact if SGRQ > 20 with n=22). Statistical analysis was performed using the two-sided Wilcoxon-rank sum test with a p < 0.05 considered significant. Data are represented as median with an interquartile range (IQR). Boxplots show minimum and maximum values, the interquartile range (IQR, 25th to 75th percentile), and the whiskers representing 1.5 times the interquartile range. Outliers are indicated as individual points outside the whiskers. Source data are provided as a Source Data file.

Supplementary Information

Supplementary Table 1. Demographic and clinical and TB-related patients' characteristics at time of surgery.

Variable	Category	Overall	Male	Female	*p*-value
N		*N*= 14	*n*= 7	*n*= 7	
**Clinical & epidemiological characteristics**					
Age [mean (SD)]		34.36 (12.02)	37 (15.56)	31 (7.39)	0.4387 [a]
BMI [median (range)]		23.55 [19.1-32]	23.4 [21.5-31-7]	23.7 [19.1-32]	0.6841 [b]
Smoker (%)	Yes	4 (28.57)	4 (57.14)	0 (0)	0.0699 [c]
	No	10 (71.43)	3 (42.86)	7 (100)	
Alcohol (%)	Yes	5 (35.71)	5 (71.43)	0 (0)	0.0210* [c]
	No	9 (64.29)	2 (28.57)	7 (100)	
Comorbidities (HCV, HBV, Diabetes) (%)	Yes	6 (42.86)	3 (42.86)	3 (42.86)	1 [c]
	No	8 (57.14)	4 (57.14)	4 (57.14)	
**Characteristics of current TB episode**					
Patient history (%)	Relapse	5 (37.71)	2 (28.57)	3 (42.86)	1 [c]
	New patient	9 (64.29)	5 (71.43)	4 (57.14)	
Drug Sensitivity (%)	DS-TB	6 (42.86)	3 (42.86)	3 (42.86)	1 [c]
	MDR/XDR-TB	8 (57.14)	4 (57.14)	4 (57.14)	
Fast (≤2 months) and slow (>2 months) time to sputum culture conversion	Fast converters	8 (57.14)	3 (42.86)	5 (71.43)	0.5921 [c]
	Slow converters	6 (42.86)	4 (57.14)	2 (28.57)	
Multiple lesions in the CXR	Yes (≥2)	4 (28.57)	2 (28.57)	2 (28.57)	1 [c]
	No (<2)	10 (71.43)	5 (71.43)	5 (71.43)	

Statistical analysis was performed by applying the two-sided: [a] t-test, [b] Mann-Whitney U-test, [c] Fisher's exact test. *Statistically significant differences refer to a p-value < 0.05 (found in alcohol use only).

Supplementary Table 2 – Transcription factors differentially expressed between clinical surrogates of treatment response and disease severity.

Gene	Module	Log2FC G vs NL	*p* adj. G vs NL	Surrogate comparison	*p* adj. surrogate comparison
ETV7	IFN/cytokine signaling	0.67	0.0120 *	SCC	0.0026 **
STAT1	IFN/cytokine signaling	0.98	0.0005 ***	SCC	0.0160 *
AR	EMT	-0.59	0.0314 *	SGRQ score	0.0013 **
STAT1	IFN/cytokine signaling	0.98	0.0005 ***	SGRQ score	0.0014 **
SOX5	EMT	-0.36	0.0079 **	SGRQ score	0.0074 **
ETV7	IFN/cytokine signaling	0.67	0.0120 *	SGRQ score	0.0079 **
ERG	EMT	-0.41	0.0311 *	SGRQ score	0.0180 *
ASCL2	IFN/cytokine signaling	1.10	0.0030 **	SGRQ score	0.0290 *
PRDM5	EMT	-0.45	0.0067 **	SGRQ score	0.0410 *

Statistical analysis was performed by applying the two-sided Wilcoxon-rank sum test. Asterisks represent statistically significant differences: *p < 0.05; **p < 0.01; ***p < 0.001. Statistical differences refer to a p-value < 0.05.
SGRQ = Saint George's Respiratory Questionnaire.
