# Supplementary material for: Unravelling the transcriptome of the human tuberculosis lesion and its clinical implications
Source: Nat Commun. 2025 May 30;16:5028. doi: 10.1038/s41467-025-60255-w (PMC12125219; doi:10.1038/s41467-025-60255-w)
Supplement: Supplementary file 2 — Description of Additional Supplementary Files [file 41467_2025_60255_MOESM2_ESM.pdf]

Description of Additional Supplementary Files

Supplementary Data 1 – Full gene expression data from RNA-seq analysis.

Supplementary Data 2 – Participants clinical data and weighted gene co-expression analysis (WGCNA).
