# Supplementary material for: Unravelling the transcriptome of the human tuberculosis lesion and its clinical implications
Source: Nat Commun. 2025 May 30;16:5028. doi: 10.1038/s41467-025-60255-w (PMC12125219; doi:10.1038/s41467-025-60255-w)
Supplement: Supplementary file 5 — Reporting Summary [file 41467_2025_60255_MOESM5_ESM.pdf]

nature portfolio

Corresponding author(s): Cristina Vilaplana

Last updated by author(s): May 2, 2025

# Reporting Summary

Nature Portfolio wishes to improve the reproducibility of the work that we publish. This form provides structure for consistency and transparency in reporting. For further information on Nature Portfolio policies, see our Editorial Policies and the Editorial Policy Checklist.

## Statistics

For all statistical analyses, confirm that the following items are present in the figure legend, table legend, main text, or Methods section.

| n/a | Confirmed | |
|---|---|---|
| ☐ | ☒ | The exact sample size (*n*) for each experimental group/condition, given as a discrete number and unit of measurement |
| ☐ | ☒ | A statement on whether measurements were taken from distinct samples or whether the same sample was measured repeatedly |
| ☐ | ☒ | The statistical test(s) used AND whether they are one- or two-sided<br>*Only common tests should be described solely by name; describe more complex techniques in the Methods section.* |
| ☒ | ☐ | A description of all covariates tested |
| ☐ | ☒ | A description of any assumptions or corrections, such as tests of normality and adjustment for multiple comparisons |
| ☐ | ☒ | A full description of the statistical parameters including central tendency (e.g. means) or other basic estimates (e.g. regression coefficient) AND variation (e.g. standard deviation) or associated estimates of uncertainty (e.g. confidence intervals) |
| ☐ | ☒ | For null hypothesis testing, the test statistic (e.g. $F$, $t$, $r$) with confidence intervals, effect sizes, degrees of freedom and $P$ value noted<br>*Give P values as exact values whenever suitable.* |
| ☒ | ☐ | For Bayesian analysis, information on the choice of priors and Markov chain Monte Carlo settings |
| ☒ | ☐ | For hierarchical and complex designs, identification of the appropriate level for tests and full reporting of outcomes |
| ☒ | ☐ | Estimates of effect sizes (e.g. Cohen's *d*, Pearson's *r*), indicating how they were calculated |

*Our web collection on statistics for biologists contains articles on many of the points above.*

## Software and code

Policy information about availability of computer code

| | |
|---|---|
| Data collection | RNA-Seq data obtention and preprocessing: Illumina platform HiSeq 4000 (Illumina), FastQC (v.011.7), MultiQC (v.1.9), Trimmomatic (v.0.39), STAR package (v.2.7.5b), Ensembl human GRCh38.89, SAMtools package (v.1.10).<br><br>Immunohistochemistry and image obtention: ultraView DAB (Ventana Medical Systems) and AxioScan 7 and Zen 3.10 imaging software (Zeiss). |
| Data analysis | RNA analysis: R (v.4.3.3), DESeq2 (v.1.40.2), pheatmap (v.1.0.12), WGCNA (v. 1.72-1), WriteXLS (v.6.4.0), readxl (v.1.4.3), gdata (v.2.19.0), genefilter (v1.82.1), ggbeeswarm (v.0.7,2), ggpubr (v.0.6.0), pheatmap (v.1.0.12), qusage (v.2.34.0), MSIgDB (v2023.1.Hs), clusterProfiler (v.4.10.1), biomaRt (v.2.58.2), tidyr (v.1.3.1), ggplot2 (v.3.5.1), org.Hs.eg.db (v.3.18.0), CytoHubba (v.3.10.2), IOBR (v 0.99.0).<br>Immunohistochemistry staining quantification analysis: ImageJ v1.54<br>Code is available at: |

For manuscripts utilizing custom algorithms or software that are central to the research but not yet described in published literature, software must be made available to editors and reviewers. We strongly encourage code deposition in a community repository (e.g. GitHub). See the Nature Portfolio guidelines for submitting code & software for further information.

# Data

Policy information about availability of data

All manuscripts must include a data availability statement. This statement should provide the following information, where applicable:

- Accession codes, unique identifiers, or web links for publicly available datasets
- A description of any restrictions on data availability
- For clinical datasets or third party data, please ensure that the statement adheres to our policy

The metadata and sequencing data generated in this study have been deposited in the National Center for Biotechnology Information Gene Expression Omnibus (GEO) database under accession code GSE184537 (https://www.ncbi.nlm.nih.gov/geo/query/acc.cgi?acc=GSE184537). The remaining data generated in this study are provided within the Article, Supplementary Information, Supplementary Data Sets and Source Data file. The patient's data used in this study are freely available in the Mendeley database under the following (doi: 10.17632/knhvdbjv3r.1). Source data are provided with this paper. The code used in this study is available on GitHub at https://doi.org/10.5281/zenodo.15322542.

# Research involving human participants, their data, or biological material

Policy information about studies with human participants or human data. See also policy information about sex, gender (identity/presentation), and sexual orientation and race, ethnicity and racism.

| | |
|---|---|
| Reporting on sex and gender | Biological sex was considered in the study design, with samples collected from 7 male and 7 female individuals. As the analysis was conducted by lung lesion compartments, stratifying by biological sex would result in a low sample size, which could limit the ability to draw meaningful conclusions. Data were disaggregated by sex where applicable (as reported in Supplementary Dataset 2), and consent for reporting and sharing individual-level data was obtained in accordance with ethical requirements. Biological sex was self-reported by study participants. |
| Reporting on race, ethnicity, or other socially relevant groupings | Socially relevant categorization variables were not included in the manuscript. |
| Population characteristics | Total of 14 TB patient: 7 females and 7 males; median age of 34.36 years old; TB drug sensitivity: 6 drug-sensitive (DS), 6 multi drug-resistant (MDR), and 2 extensively drug-resistant (XDR). Supplementary Table 1 provides the demographic, clinical, and tuberculosis-related characteristics of patients at the time of surgery. |
| Recruitment | Participants enrolled in the study were indicated for therapeutic surgery due to persistent radiological signs of cavitary lesions on Chest X-ray (CXR) and computed tomography (CT) scan, regardless of the drug-sensitivity pattern of the M. tuberculosis strain (DS- and MDR/XDR-TB), and in accordance with the official Georgian National Guideline for "Surgical Treatment of Patients with Pulmonary Tuberculosis." |
| Ethics oversight | This study is part of the SH-TBL project (ClinicalTrials.gov Identifier: NCT02715271). The protocol, research methodology and all associated documents (informed consent sheet, informed consent form) were reviewed and approved by both ethics' committees at the National Center of Tuberculosis and Lung Diseases (NCTLD) (IRB00007705 NCTLD Georgia #1, IORG0006411) and the Germans Trias i Pujol University Hospital (EC: PI-16-171). Written informed consent was obtained from all study participants before enrolment. |

Note that full information on the approval of the study protocol must also be provided in the manuscript.

# Field-specific reporting

Please select the one below that is the best fit for your research. If you are not sure, read the appropriate sections before making your selection.

☒ Life sciences ☐ Behavioural & social sciences ☐ Ecological, evolutionary & environmental sciences

For a reference copy of the document with all sections, see nature.com/documents/nr-reporting-summary-flat.pdf

# Life sciences study design

All studies must disclose on these points even when the disclosure is negative.

| | |
|---|---|
| Sample size | Sample size was not statistically determined. Instead, it was based on participant sample availability, considering participant biological sex and Mtb drug-sensitivity classification. The RNA-Seq experiments included 48 lung tissue samples from 14 patients, comprising 8 C, 13 I, 14 E, and 13 NL tissues, as detailed in Supplementary Dataset 2. |
| Data exclusions | No data excluded. |
| Replication | Not applicable |
| Randomization | Randomization was not relevant to this study because the data and samples were already collected, and there were no interventions or group assignments requiring randomization. |

| | |
|---|---|
| Blinding | Blinding was not relevant to this study because the data and samples were already collected, and there were no interventions or assessments requiring blinding. |

# Behavioural & social sciences study design

All studies must disclose on these points even when the disclosure is negative.

| | |
|---|---|
| Study description | N/A |
| Research sample | N/A |
| Sampling strategy | N/A |
| Data collection | N/A |
| Timing | N/A |
| Data exclusions | N/A |
| Non-participation | N/A |
| Randomization | N/A |

# Ecological, evolutionary & environmental sciences study design

All studies must disclose on these points even when the disclosure is negative.

| | |
|---|---|
| Study description | N/A |
| Research sample | N/A |
| Sampling strategy | N/A |
| Data collection | N/A |
| Timing and spatial scale | N/A |
| Data exclusions | N/A |
| Reproducibility | N/A |
| Randomization | N/A |
| Blinding | N/A |

Did the study involve field work? ☐ Yes ☒ No

## Field work, collection and transport

| | |
|---|---|
| Field conditions | *Describe the study conditions for field work, providing relevant parameters (e.g. temperature, rainfall).* |
| Location | *State the location of the sampling or experiment, providing relevant parameters (e.g. latitude and longitude, elevation, water depth).* |
| Access & import/export | *Describe the efforts you have made to access habitats and to collect and import/export your samples in a responsible manner and in compliance with local, national and international laws, noting any permits that were obtained (give the name of the issuing authority, the date of issue, and any identifying information).* |
| Disturbance | *Describe any disturbance caused by the study and how it was minimized.* |

# Reporting for specific materials, systems and methods

We require information from authors about some types of materials, experimental systems and methods used in many studies. Here, indicate whether each material, system or method listed is relevant to your study. If you are not sure if a list item applies to your research, read the appropriate section before selecting a response.

## Materials & experimental systems

| n/a | Involved in the study |
|---|---|
| ☐ | ☒ Antibodies |
| ☒ | ☐ Eukaryotic cell lines |
| ☒ | ☐ Palaeontology and archaeology |
| ☒ | ☐ Animals and other organisms |
| ☐ | ☒ Clinical data |
| ☒ | ☐ Dual use research of concern |
| ☒ | ☐ Plants |

## Methods

| n/a | Involved in the study |
|---|---|
| ☒ | ☐ ChIP-seq |
| ☒ | ☐ Flow cytometry |
| ☒ | ☐ MRI-based neuroimaging |

# Antibodies

Antibodies used

Antibodies used in this study:
CXCL9 (rabbit polyclonal 22355-1-AP; Proteintech, dilution 1:100)
GBP5 (rabbit polyclonal 13220-1-AP; Proteintech, dilution 1:200)
STAT1 (rabbit polyclonal 10144-2-AP; Proteintech, dilution 1:300)

Validation

All immunohistochemistry antibodies used in this study are commercially available and were validated by the manufacturer for reactivity with human samples. Additionally, all reagents were titrated and validated in-house using tonsil control samples. Lung samples from individuals who underwent surgery for bullous emphysema were used as non-TB control samples and compared to TB lung lesion samples. 13220-1-AP and 10144-2-AP have been KO validated as per manufacturer's website.

# Eukaryotic cell lines

Policy information about cell lines and Sex and Gender in Research

Cell line source(s)

*State the source of each cell line used and the sex of all primary cell lines and cells derived from human participants or vertebrate models.*

Authentication

*Describe the authentication procedures for each cell line used OR declare that none of the cell lines used were authenticated.*

Mycoplasma contamination

*Confirm that all cell lines tested negative for mycoplasma contamination OR describe the results of the testing for mycoplasma contamination OR declare that the cell lines were not tested for mycoplasma contamination.*

Commonly misidentified lines
(See ICLAC register)

*Name any commonly misidentified cell lines used in the study and provide a rationale for their use.*

# Palaeontology and Archaeology

Specimen provenance

*Provide provenance information for specimens and describe permits that were obtained for the work (including the name of the issuing authority, the date of issue, and any identifying information). Permits should encompass collection and, where applicable, export.*

Specimen deposition

*Indicate where the specimens have been deposited to permit free access by other researchers.*

Dating methods

*If new dates are provided, describe how they were obtained (e.g. collection, storage, sample pretreatment and measurement), where they were obtained (i.e. lab name), the calibration program and the protocol for quality assurance OR state that no new dates are provided.*

☐ Tick this box to confirm that the raw and calibrated dates are available in the paper or in Supplementary Information.

Ethics oversight

*Identify the organization(s) that approved or provided guidance on the study protocol, OR state that no ethical approval or guidance was required and explain why not.*

Note that full information on the approval of the study protocol must also be provided in the manuscript.

# Animals and other research organisms

Policy information about studies involving animals; ARRIVE guidelines recommended for reporting animal research, and Sex and Gender in Research

Laboratory animals

*For laboratory animals, report species, strain and age OR state that the study did not involve laboratory animals.*

| | |
|---|---|
| Wild animals | *Provide details on animals observed in or captured in the field; report species and age where possible. Describe how animals were caught and transported and what happened to captive animals after the study (if killed, explain why and describe method; if released, say where and when) OR state that the study did not involve wild animals.* |
| Reporting on sex | *Indicate if findings apply to only one sex; describe whether sex was considered in study design, methods used for assigning sex. Provide data disaggregated for sex where this information has been collected in the source data as appropriate; provide overall numbers in this Reporting Summary. Please state if this information has not been collected. Report sex-based analyses where performed, justify reasons for lack of sex-based analysis.* |
| Field-collected samples | *For laboratory work with field-collected samples, describe all relevant parameters such as housing, maintenance, temperature, photoperiod and end-of-experiment protocol OR state that the study did not involve samples collected from the field.* |
| Ethics oversight | *Identify the organization(s) that approved or provided guidance on the study protocol, OR state that no ethical approval or guidance was required and explain why not.* |

Note that full information on the approval of the study protocol must also be provided in the manuscript.

# Clinical data

Policy information about clinical studies
All manuscripts should comply with the ICMJEguidelines for publication of clinical research and a completedCONSORT checklist must be included with all submissions.

| | |
|---|---|
| Clinical trial registration | Biological specimens used in this study were obtained from individuals enrolled in the clinical study registered at ClinicalTrials.gov under the identifier NCT02715271. |
| Study protocol | https://clinicaltrials.gov/study/NCT02715271?cond=NCT02715271&rank=1 |
| Data collection | Individuals included in this project were recruited from the SH-TBL cohort, a cross-sectional study conducted at the National Center for Tuberculosis and Lung Diseases (NCTLD) in Tbilisi, Georgia, from May 2016 to May 2018. The study enrolled 40 adult patients who had received an indication for therapeutic surgery for pulmonary TB. |
| Outcomes | Primary Outcome Measures:<br>1 - Description of Clinical and Epidemiological Data of the TB patients enrolled at baseline; descriptive analysis; data recorded in a spreadsheet created ad-hoc.<br>2 - Description of the Histopathological characteristics of the TB lesions of TB patients enrolled at baseline; descriptive analysis; data recorded in a spreadsheet created ad-hoc.<br>3 - Obtention of a genic-proteomic profile of TB lesions tissues which correlate to the histopathology of the granulomas and/or the clinic-pathological features of TB patients at baseline; descriptive analysis, correlation with histopathological characteristics of TB lesions and with clinical and epidemiological data of TB patients.<br><br>Secondary Outcome Measures :<br>1 - Change in Immunological responses at baseline; and at the moment of discharge, an average of at day 15 post-enrollment. Identification of biomarkers in blood at protein level in blood and urine; validation in tissue and blood of genic biomarkers secreted (ELISA, qPCR, immunohistochemistry).<br>2 - Change in Health Quality of Life Measurements<br>measured with Health Quality of Life Questionnaires; only for Prospective Substudy |

# Dual use research of concern

Policy information about dual use research of concern

## Hazards

Could the accidental, deliberate or reckless misuse of agents or technologies generated in the work, or the application of information presented in the manuscript, pose a threat to:

| No | Yes | |
|---|---|---|
| ☐ | ☐ | Public health |
| ☐ | ☐ | National security |
| ☐ | ☐ | Crops and/or livestock |
| ☐ | ☐ | Ecosystems |
| ☐ | ☐ | Any other significant area |

## Experiments of concern

Does the work involve any of these experiments of concern:

| No | Yes | |
|---|---|---|
| ☐ | ☐ | Demonstrate how to render a vaccine ineffective |
| ☐ | ☐ | Confer resistance to therapeutically useful antibiotics or antiviral agents |
| ☐ | ☐ | Enhance the virulence of a pathogen or render a nonpathogen virulent |
| ☐ | ☐ | Increase transmissibility of a pathogen |
| ☐ | ☐ | Alter the host range of a pathogen |
| ☐ | ☐ | Enable evasion of diagnostic/detection modalities |
| ☐ | ☐ | Enable the weaponization of a biological agent or toxin |
| ☐ | ☐ | Any other potentially harmful combination of experiments and agents |

# Plants

| | |
|---|---|
| Seed stocks | N/A |
| Novel plant genotypes | N/A |
| Authentication | N/A |

# ChIP-seq

## Data deposition

☐ Confirm that both raw and final processed data have been deposited in a public database such as GEO.

☐ Confirm that you have deposited or provided access to graph files (e.g. BED files) for the called peaks.

| | |
|---|---|
| Data access links<br>*May remain private before publication.* | *For "Initial submission" or "Revised version" documents, provide reviewer access links. For your "Final submission" document, provide a link to the deposited data.* |
| Files in database submission | *Provide a list of all files available in the database submission.* |
| Genome browser session<br>(e.g. UCSC) | *Provide a link to an anonymized genome browser session for "Initial submission" and "Revised version" documents only, to enable peer review. Write "no longer applicable" for "Final submission" documents.* |

## Methodology

| | |
|---|---|
| Replicates | *Describe the experimental replicates, specifying number, type and replicate agreement.* |
| Sequencing depth | *Describe the sequencing depth for each experiment, providing the total number of reads, uniquely mapped reads, length of reads and whether they were paired- or single-end.* |
| Antibodies | *Describe the antibodies used for the ChIP-seq experiments; as applicable, provide supplier name, catalog number, clone name, and lot number.* |
| Peak calling parameters | *Specify the command line program and parameters used for read mapping and peak calling, including the ChIP, control and index files used.* |
| Data quality | *Describe the methods used to ensure data quality in full detail, including how many peaks are at FDR 5% and above 5-fold enrichment.* |
| Software | *Describe the software used to collect and analyze the ChIP-seq data. For custom code that has been deposited into a community repository, provide accession details.* |

# Flow Cytometry

## Plots

Confirm that:

☐ The axis labels state the marker and fluorochrome used (e.g. CD4-FITC).

☐ The axis scales are clearly visible. Include numbers along axes only for bottom left plot of group (a 'group' is an analysis of identical markers).

☐ All plots are contour plots with outliers or pseudocolor plots.

☐ A numerical value for number of cells or percentage (with statistics) is provided.

## Methodology

| | |
|---|---|
| Sample preparation | *Describe the sample preparation, detailing the biological source of the cells and any tissue processing steps used.* |
| Instrument | *Identify the instrument used for data collection, specifying make and model number.* |
| Software | *Describe the software used to collect and analyze the flow cytometry data. For custom code that has been deposited into a community repository, provide accession details.* |
| Cell population abundance | *Describe the abundance of the relevant cell populations within post-sort fractions, providing details on the purity of the samples and how it was determined.* |
| Gating strategy | *Describe the gating strategy used for all relevant experiments, specifying the preliminary FSC/SSC gates of the starting cell population, indicating where boundaries between "positive" and "negative" staining cell populations are defined.* |

☐ Tick this box to confirm that a figure exemplifying the gating strategy is provided in the Supplementary Information.

# Magnetic resonance imaging

## Experimental design

| | |
|---|---|
| Design type | *Indicate task or resting state; event-related or block design.* |
| Design specifications | *Specify the number of blocks, trials or experimental units per session and/or subject, and specify the length of each trial or block (if trials are blocked) and interval between trials.* |
| Behavioral performance measures | *State number and/or type of variables recorded (e.g. correct button press, response time) and what statistics were used to establish that the subjects were performing the task as expected (e.g. mean, range, and/or standard deviation across subjects).* |

## Acquisition

| | |
|---|---|
| Imaging type(s) | *Specify: functional, structural, diffusion, perfusion.* |
| Field strength | *Specify in Tesla* |
| Sequence & imaging parameters | *Specify the pulse sequence type (gradient echo, spin echo, etc.), imaging type (EPI, spiral, etc.), field of view, matrix size, slice thickness, orientation and TE/TR/flip angle.* |
| Area of acquisition | *State whether a whole brain scan was used OR define the area of acquisition, describing how the region was determined.* |

Diffusion MRI ☐ Used ☐ Not used

## Preprocessing

| | |
|---|---|
| Preprocessing software | *Provide detail on software version and revision number and on specific parameters (model/functions, brain extraction, segmentation, smoothing kernel size, etc.).* |
| Normalization | *If data were normalized/standardized, describe the approach(es): specify linear or non-linear and define image types used for transformation OR indicate that data were not normalized and explain rationale for lack of normalization.* |
| Normalization template | *Describe the template used for normalization/transformation, specifying subject space or group standardized space (e.g. original Talairach, MNI305, ICBM152) OR indicate that the data were not normalized.* |
| Noise and artifact removal | *Describe your procedure(s) for artifact and structured noise removal, specifying motion parameters, tissue signals and physiological signals (heart rate, respiration).* |

Volume censoring | *Define your software and/or method and criteria for volume censoring, and state the extent of such censoring.*

## Statistical modeling & inference

Model type and settings | *Specify type (mass univariate, multivariate, RSA, predictive, etc.) and describe essential details of the model at the first and second levels (e.g. fixed, random or mixed effects; drift or auto-correlation).*

Effect(s) tested | *Define precise effect in terms of the task or stimulus conditions instead of psychological concepts and indicate whether ANOVA or factorial designs were used.*

Specify type of analysis: ☐ Whole brain ☐ ROI-based ☐ Both

Statistic type for inference | *Specify voxel-wise or cluster-wise and report all relevant parameters for cluster-wise methods.*

(See Eklund et al. 2016)

Correction | *Describe the type of correction and how it is obtained for multiple comparisons (e.g. FWE, FDR, permutation or Monte Carlo).*

## Models & analysis

| n/a | Involved in the study |
|---|---|
| ☐ | ☐ Functional and/or effective connectivity |
| ☐ | ☐ Graph analysis |
| ☐ | ☐ Multivariate modeling or predictive analysis |

Functional and/or effective connectivity | *Report the measures of dependence used and the model details (e.g. Pearson correlation, partial correlation, mutual information).*

Graph analysis | *Report the dependent variable and connectivity measure, specifying weighted graph or binarized graph, subject- or group-level, and the global and/or node summaries used (e.g. clustering coefficient, efficiency, etc.).*

Multivariate modeling and predictive analysis | *Specify independent variables, features extraction and dimension reduction, model, training and evaluation metrics.*
